# Development and validation of the BMQ-AIR^©^: a screening tool for assessing patients’ treatment beliefs about switching to anti-inflammatory reliever (AIR) therapy

**DOI:** 10.3389/fphar.2024.1351851

**Published:** 2024-06-28

**Authors:** Holly Foot, Amy Hai Yan Chan, Rob Horne

**Affiliations:** ^1^ School of Pharmacy, The University of Queensland, Brisbane, QLD, Australia; ^2^ School of Pharmacy, The University of Auckland, Auckland, New Zealand; ^3^ Centre of Behavioural Medicine, School of Pharmacy, University College London, London, United Kingdom; ^4^ International Primary Care Respiratory Group, London, United Kingdom

**Keywords:** asthma, anti-inflammatory reliever therapy, reliever, short-acting beta-2 agonists, inhaled corticosteroid, questionnaire, screening tool

## Abstract

**Introduction:**

Despite anti-inflammatory reliever (AIR) therapy now being the preferred treatment choice across all severities of asthma, many patients are still “attached” to their short-acting beta^2^-agonist (SABA) reliever, believing this to be the best way to control their asthma. To encourage individuals to switch to AIR, it is important to first identify the beliefs that patients hold about AIR.

**Objective:**

The aim of this paper was to describe the initial development and validation of the BMQ-AIR^©^, a six-item screening tool which assesses and identifies patients’ treatment beliefs about switching to AIR therapy.

**Methods:**

Statements were identified from the primary literature that assessed patients’ perceptions of AIR therapy and adapted from the Beliefs about Medicines Questionnaire (BMQ). Internal reliability was examined using Cronbach’s alpha coefficient. Construct validity was evaluated by comparing scores on BMQ-AIR^©^ with a validated measure of medication adherence and SABA beliefs.

**Results:**

A total of 446 participants completed the online survey. The BMQ-AIR^©^ contained two subscales with three items each. Both the Necessity and Concerns subscales demonstrated good internal reliability, with Cronbach’s α-values of 0.70 and 0.69, respectively. Both subscales were negatively correlated with self-report inhaled corticosteroid adherence (Necessity: r = −0.28, *p* < 0.0001; Concerns: r = −0.28, *p* < 0.0001) and positively correlated with SRQ scores (Necessity: r = 0.51, *p* < 0.0001; Concerns: r = 0.44, *p* < 0.0001).

**Conclusion:**

Preliminary findings indicate that BMQ-AIR^©^ demonstrates satisfactory reliability and validity. BMQ-AIR^©^ is a promising tool that may help tailor interventions to an individual’s specific beliefs and barriers to switching to better support individuals in stopping SABA and initiating AIR therapy.

## 1 Introduction

Asthma has traditionally been managed with a preventer, usually containing an inhaled corticosteroid (ICS), and a reliever containing a short-acting beta^2^-agonist (SABA) (FitzGerald et al., 2017). Despite these medicines being efficacious, many patients overuse their SABA and underuse their ICS, and this has been shown to lead to worse asthma outcomes, including hospitalization and death (FitzGerald et al., 2017; Nwaru et al., 2020). Because of this, in the most recent treatment guidelines, there is now a shift in recommendations across all severities of asthma for patients to use anti-inflammatory reliever (AIR) therapy in place of their SABA inhaler for symptom relief (Global Initiative for Asthma, 2019). This is particularly focused on individuals whose asthma is not well-controlled by their existing treatment. AIR therapy is the use of an inhaler containing an ICS in combination with a fast-onset long-acting beta-agonist (formoterol) when required for symptom control. AIR therapy has been shown to provide better asthma symptom control and reduce severe asthma exacerbations, compared to SABA, while having an equivalent onset of action (Boonsawat et al., 2003; OByrne et al., 2018; Beasley et al., 2019; Hardy et al., 2019).

The change from SABA to AIR therapy for asthma management represents a significant change for many people with asthma. For conversion to AIR therapy to be successful, a change in two behaviors is required: individuals need to stop taking their SABA and initiate AIR therapy. This may require health professionals to identify and address potentially misplaced beliefs in their patients about the importance of SABA and concerns about ICS. Many patients are “attached” to their SABA reliever, believing this to be the best way to control their asthma (Cole et al., 2013; Reddel et al., 2017). In addition, individuals may have concerns regarding starting AIR therapy, particularly as they contain “steroids.” Concerns about steroids are common in individuals with asthma and relate to worries about the long-term effects of steroids, dependency on steroids, and side effects (Cooper et al., 2015; Chapman et al., 2017). These concerns can lead to non-adherence, even when the recommendation is based on evidence-based guidelines (Chapman et al., 2017). Convincing patients to make such a fundamental change may require discussions with health professionals in a way that addresses the individual’s beliefs about the necessity of their SABA in relation to changing to AIR therapy and addresses any concerns they may have about steroids (Østrem and Horne, 2015).

The first step to effective conversations to encourage individuals to switch to AIR is to identify the beliefs that patients hold about AIR. There is currently no screening tool available to identify the beliefs patients have about AIR therapy. The Beliefs about Medicines Questionnaire (BMQ) is a valid and reliable measure of patients’ beliefs about treatment that has been widely used in asthma in numerous countries (Horne et al., 1999; Horne and Weinman, 2002; Menckeberg et al., 2008; Chapman et al., 2017; Foot et al., 2017). The BMQ was developed based on the Necessity–Concerns Framework, which states that an individual’s adherence to their medicines is influenced by beliefs about the necessity and concerns of their treatment (Horne and Weinman, 1999; Horne et al., 2013). This is likely to apply to patients’ beliefs about switching to AIR therapy. Identifying the beliefs patients have about switching to AIR treatment will also help ensure future interventions are responsive to these individual patient beliefs and barriers.

The aim of this paper is to describe the initial development and validation of the BMQ-AIR^©^, a screening tool which assesses and identifies patients’ treatment beliefs about switching to AIR therapy.

## 2 Methods

### 2.1 Item development of the BMQ-AIR^©^

Statements were identified from the primary literature that assessed patients’ perceptions of AIR therapy and adapted from the Necessity and Concerns subscales of the BMQ (Horne et al., 1999). The BMQ is a widely used and validated tool designed to measure patients’ beliefs about the necessity and concerns of their treatment. The statements in the 10-item BMQ Necessity and Concern subscales were initially adapted to generate six items that reflected the personal need and concerns about AIR therapy. The statements were chosen to reflect the beliefs likely to be associated with perceived need and concerns about AIR and from consensus discussions with the International Primary Care Respiratory Group. These statements were then reviewed by a multidisciplinary expert panel. AIR therapy was described to participants as inhalers that combine preventer and reliever medication in one, which can be used as a reliever as needed, as well as a regular preventer.

In accordance with the BMQ scoring, each item was scored on a five-point Likert scale with 1 = strongly disagree and 5 = strongly agree. The Necessity subscale (items 1, 2, and 3) scoring ranged from 3 to 15, with higher scores indicating stronger beliefs in the necessity of AIR compared to their current asthma therapy. The Concerns subscale (items 5, 6, and 7) scoring ranged from 3 to 15, with higher scores indicating stronger beliefs about the concerns of AIR, particularly about ICS concerns.

### 2.2 Evaluating the reliability and validity of the BMQ-AIR^©^

#### 2.2.1 Participant population

Participants were recruited using the Amazon Mechanical Turk (mTurk) platform, an online participant recruitment portal where participants are invited to complete tasks requiring human involvement and are reimbursed with small monetary rewards. This online recruitment platform has been reported to be as reliable as conventional recruitment methods, and responses collected are comparable to those collected using more conventional methods (Mortensen and Hughes, 2018). A number of methods have been proposed to help ensure response quality (Aguinis et al., 2021). In this study, all responses underwent a quality review, which was based on the IP address (duplicates removed), rating quality on mTurk (low-quality ratings removed), and an open-ended question regarding asthma triggers (irregular/abnormal responses removed).

Participants self-selected questionnaire completion by responding to the online survey link posted on the mTurk platform and completed a set of screening questionnaires to confirm study’s inclusion eligibility. To be eligible to participate, participants needed to self-report a diagnosis of asthma and be at least 18 years old. Participants were reimbursed US$3 for the completion of the survey. According to an online review by the UK NHS Research Ethics Committee, no further ethical approval was deemed necessary for this study as no identifiable data were collected from individuals, and all data were anonymized.

#### 2.2.2 Item analysis

Descriptive analyses were conducted on participants’ responses to describe the means, standard deviations, and frequency distributions of each BMQ-AIR^©^ item. Frequency distributions for both Necessity and Concern subscales were also calculated. This was based on the participants’ mean BMQ-AIR^©^ subscale scores, calculated by adding the score for each item, then dividing by the number of items 3) to produce a mean overall score between 1 and 5. Item analysis also identified the percentage of participants who responded strongly disagree/disagree/uncertain to each of the Necessity subscale items and agree/strongly agree to each of the Concern subscale items.

Participants were also categorized into attitudinal groups (skeptical, ambivalent, indifferent, and accepting groups) based on whether they scored above or below/equal to the scale midpoint 9) for the Necessity and Concerns subscale of the BMQ-AIR^©^.

#### 2.2.3 Internal reliability analysis

To assess the internal reliability of BMQ-AIR^©^, Cronbach’s α-values for each of the subscales were calculated. Cronbach’s α was also calculated for remaining items when each item was deleted one at a time to evaluate each item’s contribution to the internal consistency reliability of the BMQ-AIR^©^.

#### 2.2.4 Validity testing

Validity relates to evaluating whether the questionnaire measures what it intends to measure, i.e., beliefs about the necessity and concerns of AIR therapy. There is currently no standard criterion (i.e., gold standard) for measuring patients’ beliefs about AIR therapy. Therefore, construct validity for the BMQ-AIR^©^ was evaluated by exploring Pearson’s correlations between BMQ-AIR^©^ subscale scores for the Necessity and Concerns subscales: 1) a self-report measure of adherence to ICS (measured by the Medication Adherence Report Scale [MARS]) (Horne and Weinman, 2002; Chan et al., 2020a) and 2) beliefs in the necessity of reliever therapy (measured by the SABA Reliance Questionnaire [SRQ] (Chan et al., 2020b)).

The nine-item MARS was used to assess medication-taking behaviors related to participants’ use of ICS preventer medication. Each of the nine items related to a medication-taking behavior and was rated on a five-point Likert scale, from always (1) to never (5). Higher scores indicate better adherence. To assess patients’ perceived need for their current reliever inhaler, the SRQ was used. The SRQ is a five-item scale, with each of the items scored on a five-point Likert scale with 1 = strongly disagree and 5 = strongly agree. Total scores ranged from 5 to 25, with higher scores indicating higher necessity beliefs for SABA (i.e., higher reliance on SABA).

These criteria were chosen based on the hypothesis that relationships between medication beliefs and medication-taking behavior in asthma are primarily driven by perceived concerns about steroids (Cooper et al., 2015; Chapman et al., 2017). These concerns can contribute to ICS preventer non-adherence and SABA over-reliance as SABA inhalers do not contain ICS and may fundamentally be perceived as the “safer” inhaler between traditional preventers vs. reliever inhalers (Tavakoli et al., 2018; Blakeston et al., 2021). At the same time, we hypothesize that individuals who have concerns about steroids are likely to prefer treatments that lead to overall reduced steroid exposure, which may result in perceived increased need for AIR therapy. Although AIR does contain ICS, AIR only needs to be used intermittently rather than on a regular basis. For individuals who are concerned about steroid exposure, AIR therapy may provide a more appealing option for these individuals, who may be non-adherent to daily ICS preventers due to concerns around steroids. Using a “when required” ICS-containing preventer may appear more attractive than having to use ICS regularly, and therefore, such individuals may perceive a higher need for AIR therapy over current therapy (i.e., over regular ICS + SABA treatment). As such, the following hypotheses were used to test the validity of the BMQ-AIR^©^ Necessity and Concerns subscales.

##### 2.2.4.1 Necessity subscale of the BMQ-AIR^©^

The following hypotheses were used to test the construct validity of the Necessity subscale of the BMQ-AIR^©^:

- Self-reported adherence to ICS preventative therapy

Individuals who are poorly adherent to their current ICS preventative therapy are more likely to want to switch or change treatments (i.e., to AIR therapy). Therefore, we hypothesized that a low MARS score (indicating poor ICS adherence) would be associated with high Necessity scores on the BMQ-AIR^©^ (i.e., negatively correlated).

- Reliance on current reliever therapy

It was hypothesized that individuals who rely on their reliever are more likely to also perceive a strong need for the alternative reliever (i.e., AIR therapy). The SRQ indicates necessity for the reliever; as such, we hypothesized that high SRQ scores would be related to high Necessity scores on the BMQ-AIR^©^ (i.e., positively correlated).

##### 2.2.4.2 Concerns subscale of the BMQ-AIR^©^

The following hypotheses were used to test the construct validity of the Concern subscale of the BMQ-AIR^©^:

- Self-reported adherence to ICS preventative therapy

Perceived concerns about steroid (i.e., the ICS component of AIR) treatment are likely to be higher in individuals who have pre-existing concerns about their ICS preventer inhaler. This is likely to manifest as ICS non-adherence. It was hypothesized that patients who had high existing concerns about their current ICS treatment would also have concerns about the steroid (ICS) component of AIR therapy. As such, we hypothesized that low MARS scores (i.e., low ICS adherence) would be associated with high concerns on the BMQ-AIR^©^ (i.e., negatively correlated).

- Necessity of current reliever therapy

Individuals, who have concerns about steroids and thus correspondingly low ICS adherence, are more likely to prefer using their SABA over their ICS inhaler. This may inadvertently lead to the SABA over-reliance (Chan et al., 2020b; Blakeston et al., 2021). It was hypothesized that high concerns subscale scores on BMQ-AIR^©^, which may be driven by concerns about the ICS within the AIR therapy, would be associated with higher reliance on SABA, as indicated by high SRQ scores (i.e., positively correlated).

## 3 Results

A total of 446 participants completed the mTurk survey. The BMQ-AIR^©^ contained six items: three Necessity items and three Concern items.

### 3.1 Univariate analysis of scale items

#### 3.1.1 Means and standard deviations

The means and standard deviations (SDs) of the participants’ scores for each of the items on the BMQ-AIR^©^ are shown in Table 1. Higher scores indicate a stronger perceived need or concern about AIR therapy.

**TABLE 1:** Means and standard deviations (SDs) of each item of the six-item BMQ-AIR^©^.

Scale item *NB: Item wording is copyrighted to Professor Rob Horne–please contact Professor Horne for permission to reuse	Mean	±SD
Necessity subscale (scored 1–5, higher scores indicate stronger agreement with the statements)		
1. I would be happy to replace my reliever with an anti-inflammatory reliever (N1)	3.59	0.98
2. Using an anti-inflammatory reliever would be more effective for managing my asthma than my preventer and reliever separately (N2)	3.59	1.02
3. If I were having an asthma attack, I would not worry about not having my reliever inhaler as long as I had my anti-inflammatory reliever (N3)	3.50	1.07
Concerns subscale (scored 1–5, higher scores indicate stronger agreement with the statements)		
4. I do not like the idea of taking a corticosteroid more often (C1)	3.54	1.05
5. I worry about the long-term effects of an anti-inflammatory reliever (C2)	3.61	1.05
6. I am concerned that an anti-inflammatory reliever would be less safe than my usual reliever (C3)	3.55	1.08

#### 3.1.2 Frequency distributions

**FIGURE 1:**
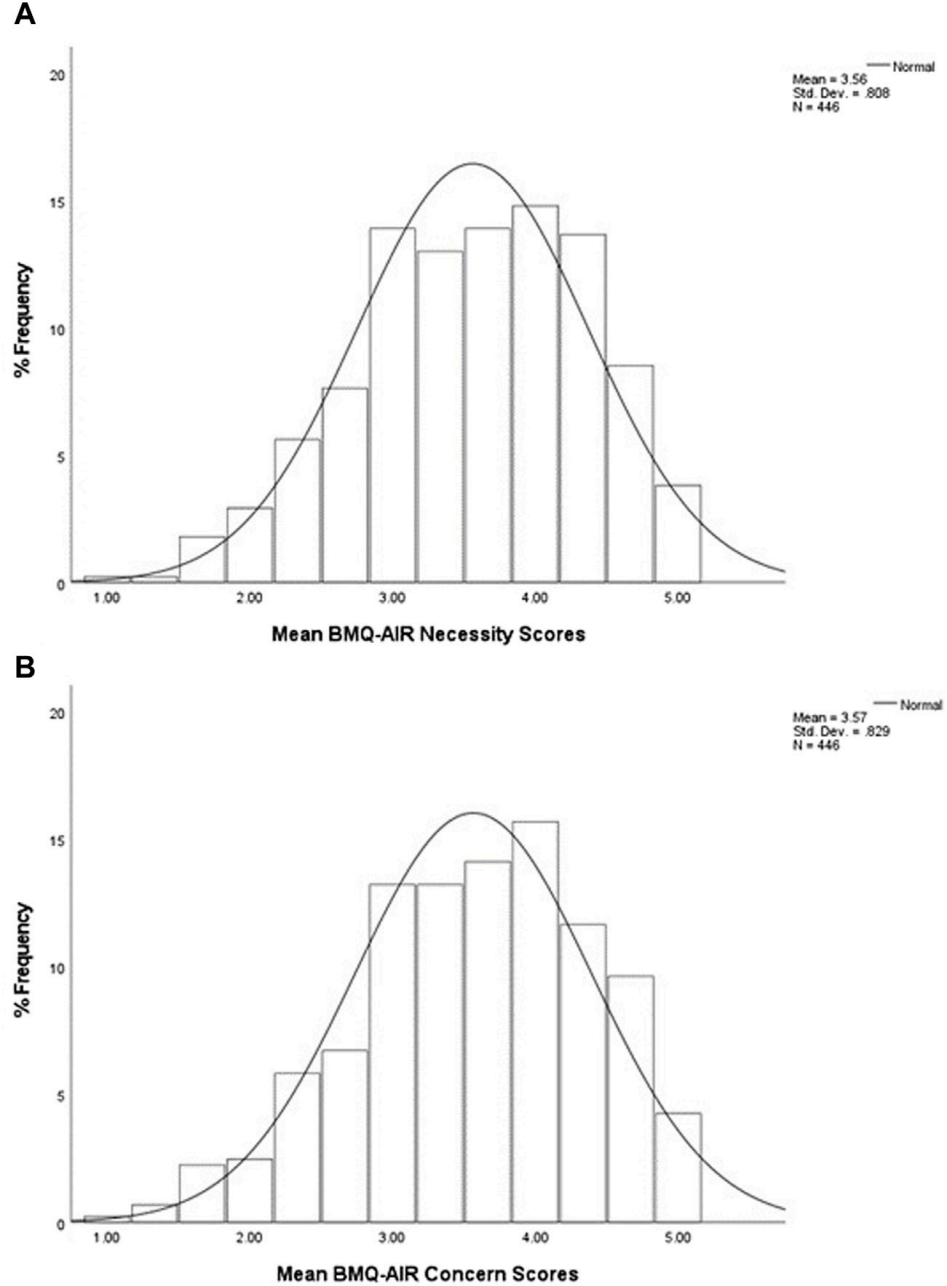
Percentage frequency distributions of participants’ mean scores to **(A)** Necessity subscale and **(B)** Concerns subscale, on a five-point Likert scale (*n* = 446). The mean (±SD) scores for the sample population were 3.56 (0.81) for the Necessity subscale and 3.57 (0.83) for the Concerns subscale. This indicates that when considering participants’ overall subscale scores, more participants chose agree/strongly agree responses to both subscale items.

The frequency distributions of participants’ mean scores for the Necessity and Concern subscales are shown in Figure 1.

#### 3.1.3 Item analysis

**FIGURE 2:**
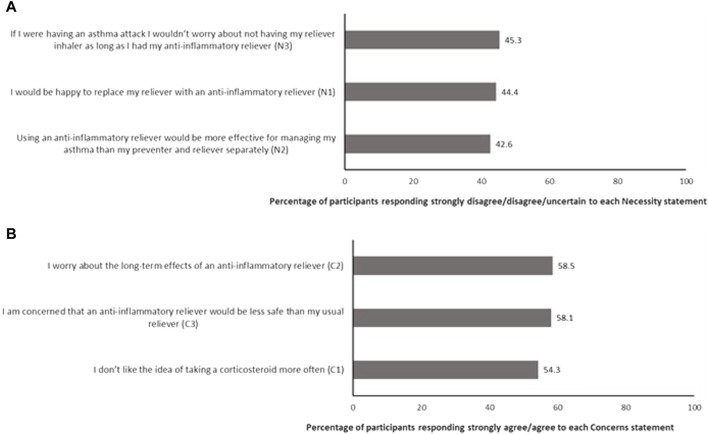
Item analysis of the BMQ-AIR^©^ describing percentage of participants who **(A)** have doubts about the necessity of AIR and **(B)** have concerns about AIR (*n* = 446).

The BMQ-AIR^©^ item analysis is shown in Figure 2. The percentage of participants who had doubts about the necessity of AIR, defined as those that responded strongly disagree, disagree, or uncertain to each of the three Necessity subscale items, is shown in Figure 2A. Conversely, the percentage of participants who held concerns about AIR, defined as those that responded strongly agree or agree to each of the three Concerns subscale items of the BMQ-AIR^©^, is shown in Figure 2B. As shown in the figure, an agreement with most concern statements was high. Item 2 (worry about long-term effects) of the Concerns subscale was the item that most participants agreed or strongly agreed with (58.5%). In contrast, item 1 (do not like taking corticosteroid more often) of the Concerns subscale had fewer agree/strongly agree responses, but still, more than half the sample agreed/strongly agreed with this statement.

**FIGURE 3:**
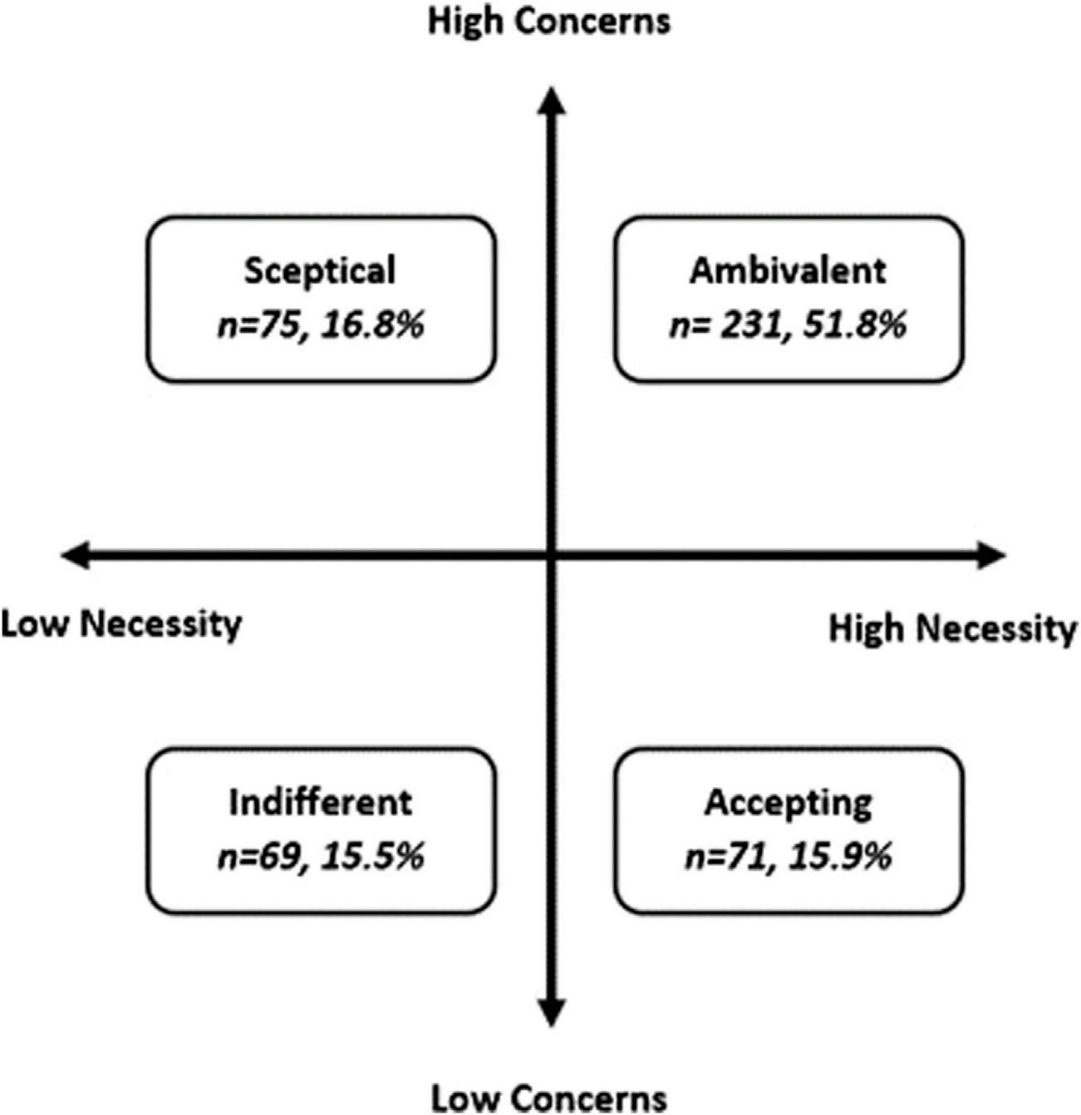
Attitudinal analysis of participants’ beliefs about AIR therapy (*n* = 446).

When the Necessity and Concerns scores were combined in an attitudinal analysis, over half of the participants were classed as “ambivalent” toward AIR therapy (51.8%, *n* = 231) (Figure 3), defined as higher-than-midpoint necessity and concern subscale scores. The remaining participants were split almost equally as being “accepting” (15.9%, *n* = 71), “skeptical” (16.8%, *n* = 75), or “indifferent” (15.5%, *n* = 69).

### 3.2 Internal reliability analysis

Both subscales of BMQ-AIR^©^ demonstrated good internal reliability for a three-item scale. The Necessity and Concerns subscales had Cronbach’s α values of 0.70 and 0.69, respectively. As shown in Table 2, the internal reliability did not improve by removing further scale items.

**TABLE 2:** Cronbach’s α coefficient of scale if the scale item is deleted.

Scale item *NB: Item wording is copyrighted to professor Rob Horne–please contact professor Horne for permission to reuse	Cronbach’s α if statement removed
Necessity subscale	
1. I would be happy to replace my reliever with an anti-inflammatory reliever (N1)	0.61
2. Using an anti-inflammatory reliever would be more effective for managing my asthma than my preventer and reliever separately (N2)	0.58
3. If I were having an asthma attack, I would not worry about not having my reliever inhaler as long as I had my anti-inflammatory reliever (N3)	0.62
Concerns subscale	
4. I do not like the idea of taking a corticosteroid more often (C1)	0.61
5. I worry about the long-term effects of an anti-inflammatory reliever (C2)	0.60
6. I am concerned that an anti-inflammatory reliever would be less safe than my usual reliever (C3)	0.57

### 3.3 Construct validity

#### 3.3.1 Necessity subscale

In line with the study hypotheses, Necessity subscale scores were negatively correlated with self-reported ICS adherence (r = −0.28, *p* < 0.0001) and positively correlated with SRQ scores (r = 0.51, *p* < 0.0001). Participants who reported a strong need to switch to AIR therapy were also likely to be less adherent to their current ICS preventer and more likely to rely on their current reliever.

#### 3.3.2 Concerns subscale

Similarly, BMQ-AIR^©^ concerns subscale scores were negatively correlated with self-reported ICS adherence (r = −0.28, *p* < 0.0001) and positively correlated with SRQ scores (r = 0.44, *p* < 0.0001). Participants who have high perceived concerns about switching to AIR therapy were those who were less likely to be adherent to their current ICS preventer and more likely to rely on their current reliever.

## 4 Discussion

This paper reports on the development and validation of BMQ-AIR^©^ for use in individuals with asthma, who are considering a switch to AIR therapy from their traditional ICS preventer and SABA reliever regimen. BMQ-AIR^©^ is a novel tool designed to elicit patients’ beliefs about AIR therapy which may be used to guide effective conversations between health professionals and patients and inform strategies to encourage individuals to switch from traditional ICS and SABA therapy to AIR therapy. Switching patients to AIR therapy has been proven in several clinical trials to be superior in terms of symptom control while reducing the frequency of severe asthma exacerbations compared to the use of SABA monotherapy as a reliever treatment (OByrne et al., 2018; Beasley et al., 2019; Hardy et al., 2019).

Although it is now a gold standard across all severity levels of asthma to be prescribed AIR therapy in favor of SABA (Global Initiative for Asthma, 2019), switching from SABA to AIR therapy can be challenging. Patients are often attached to their SABA and also have concerns about inhalers which contain steroids.^9,10 23^ Interestingly, patients’ perceived concerns about steroids are more likely to relate to non-adherence than their actual experience of side effects (Cooper et al., 2015). In the present study, as demonstrated through the attitudinal analysis, while individuals may indicate a perceived need for a new reliever treatment, they were also concerned about the new therapy, which is likely linked to the perceived concerns about steroids (ICS). Over half the cohort was identified as being ambivalent toward AIR therapy. This demonstrates the importance of identifying both necessity and concern beliefs in an individual as both are likely to play a role in influencing an individual’s decision to switch, or not to switch, to AIR therapy. Understanding each of the four attitudinal beliefs may be useful for guiding consultations and discussions with health professionals as the adherence behaviors of people within each of the four categories are often different. For example, a study in stroke, rheumatoid arthritis, and diabetes showed that, compared with patients in the accepting group, patients in the other attitudinal groups were more likely to be non-adherent (Wei et al., 2017). For patients with coexisting necessity and concern beliefs about AIR therapy, one strategy to encourage patients to switch to AIR therapy is to inform patients that side effects with ICS are rare and mild and that the AIR therapy strategy leads to a lower total daily steroid dose as it only treats inflammation when it is present rather than needing to use steroids every day regularly (Kaplan et al., 2020).

At present, there is no specific tool designed to elicit patient’s beliefs and attitudes about AIR therapy; currently, patients’ attitudes and beliefs can only be assessed through the use of general questionnaires, such as the BMQ, or through unstructured conversations that may not identify the specific concerns patients hold about switching to AIR. The original BMQ is designed to assess beliefs about an individual’s current medication regimen rather than future or alternate medication options and is therefore not appropriate for use in patients who are not yet on AIR therapy but are considering a switch to AIR. SRQ can help identify patients who may be overusing SABA, but as it is designed specifically to focus on SABA treatment, it cannot identify the beliefs patients hold about AIR. Although healthcare professionals have been urged to change patients from SABA to AIR therapy (Levy et al., 2024), a specific tool to assess beliefs about AIR is needed. The BMQ-AIR^©^ was developed from the framework behind the original BMQ, which proposes that individuals weigh up decisions about treatment using a Necessity–Concerns Framework, considering their beliefs about the necessity of the medication for maintaining health (specific necessity) and concerns about side effects and harm from the medication (specific concerns).

BMQ-AIR^©^ is the first questionnaire designed to elicit beliefs around switching to AIR therapy. It has shown good construct validity and internal reliability in this initial validation study in 446 individuals with self-reported asthma. As there are no other questionnaires that measure beliefs about AIR therapy, the validation of the tool was demonstrated through significant relationships with reliance on SABA and adherence to current ICS therapy. We acknowledge that the magnitude of the association between adherence and AIR therapy beliefs is weak; however, they are comparable to the relationship reported between the studies examining the relationship between the BMQ and measures of adherence in other conditions (Horne et al., 2013; Foot et al., 2016). Interestingly, the correlations between BMQ-AIR^©^ subscales and reliever reliance were stronger than those with ICS adherence. This suggests that reliance on SABA relievers is a stronger predictor of AIR beliefs than current adherence to ICS preventers.

BMQ-AIR^©^ is currently limited to being validated in an online sample of participants who self-reported asthma, and the intention to switch to AIR in the sample was not assessed. Although patients were not explicitly asked whether they were prescribed an ICS, it was made clear in the survey that the questions were for those taking reliever and controller medication. Although all participants answered the self-reported ICS adherence and the histogram of responses was as expected, we cannot be completely sure that all participants were on an ICS. A challenge of the study was the lack of a gold-standard measure for comparison. The chosen tools were used as the best measures available, and it is important to continue to examine the relationship between BMQ-AIR and measures of asthma-treatment beliefs in future studies. Furthermore, it would be useful for future research to consider assessing the effects of using different midpoints of the necessity concerns attitudinal analysis and how this may affect the results.

Although this limits the current interpretation of the results, this tool shows promise and forms the foundation for further research. Further testing should be conducted in another asthma population, potentially those seeking SABA in community pharmacies. The study design was chosen due to its cost-effective nature and its ability to recruit a diverse sample of participants. This recruitment strategy was also used in the development of the SABA Reliance Questionnaire (SRQ) (Chan et al., 2020b), which has since produced comparable results in studies utilizing more traditional methods of recruitment (Guyton and Jackson, 2022; Martínez et al., 2023). Future work is needed to confirm BMQ-AIR validity, specifically investigating the utility of the BMQ-AIR^©^ in clinician-diagnosed asthma and its relationship with actual switch rates from SABA to AIR therapy.

This study has described a novel questionnaire that assesses and identifies patients’ treatment beliefs about switching to AIR therapy. Preliminary findings indicate that BMQ-AIR^©^ demonstrates satisfactory initial reliability and validity. The implementation of BMQ-AIR^©^ into clinical practice may help enhance patient-centered care by directing health professionals to potentially misplaced beliefs about concerns about ICS. This can help ensure future interventions are responsive to an individual’s specific beliefs and barriers to switching to better support individuals in stopping their SABA and initiating AIR therapy.

## Data Availability

The raw data supporting the conclusion of this article will be made available by the authors, without undue reservation.
